# Neutrophil Extracellular Traps Promote AIM2-Dependent Microglial Pyroptosis Following Stroke

**DOI:** 10.14336/AD.2024.1733

**Published:** 2026-05-30

**Authors:** Hanze Chen, Linhui Ni, Jinhua Zhang, Xu Zheng, Yigang Chen, Xing Jin, Beibei Hu, Xinxin Xu, Qiwen Tang, Shuang Li, Yonggang Hao, Shilong Sun, Chengbin He, Shuxia Cao, Xingyue Hu

**Affiliations:** ^1^Department of Neurology, Sir Run Run Shaw Hospital, School of Medicine, Zhejiang University, Hangzhou, China.; ^2^Department of Neurology, The Fourth Affiliated Hospital of Soochow University, Suzhou, China.; ^3^Department of Ultrasound, Sir Run Run Shaw Hospital, School of Medicine, Zhejiang University, Hangzhou, China.; ^4^Department of Vascular Surgery, The First Affiliated Hospital, School of Medicine, Zhejiang University, Hangzhou, China.; ^5^Department of Radiology, Sir Run Run Shaw Hospital, School of Medicine, Zhejiang University, Hangzhou, China.

**Keywords:** Acute ischemic stroke, Ischemia-reperfusion, Neutrophil extracellular traps, Absent in melanoma 2, Pyroptosis

## Abstract

Neutrophils are among the earliest and most abundant immune cells infiltrating the brain following ischemic stroke, aggravating neuroinflammation through the formation of neutrophil extracellular traps (NETs). Pyroptosis, an inflammasome-mediated form of programmed cell death, occurs in post-stroke brain tissue and amplifies inflammation by releasing proinflammatory mediators, propagating the inflammatory cascade. However, the mechanistic link between NETs and pyroptosis remains unclear. This study demonstrated significantly elevated NET levels in arterial blood at the infarct site compared with venous or femoral arterial blood in stroke patients. A positive correlation was observed between the 24-h change in NIHSS score (NIHSS_baseline_ − NIHSS_24h_) and the difference in arterial citrullinated histone 3 (CitH3)-DNA (NETs) levels between the infarct site and femoral artery (NETs_infarct site_ − NETs_femoral artery_). In a murine stroke model, NETs were detected in the brain parenchyma. Pharmacological inhibition of NET formation with GSK484, a selective protein-arginine deiminase type 4 antagonist, suppressed NET production, reduced absent in melanoma 2 (AIM2) inflammasome expression, and improved neurological outcomes in mice following stroke. AIM2 knockdown in brain tissue achieved similar neuroprotective effects. In both BV2 cells and stroke-induced mice, NETs triggered AIM2-dependent pyroptosis. These findings suggest that neutrophils in peripheral blood infiltrate the brain parenchyma to generate NETs, activating the AIM2 inflammasome in microglia and exacerbating stroke-induced brain injury through pyroptosis. Targeting NET formation or AIM2 inflammasome activation represents a potential therapeutic strategy for attenuating post-stroke neuroinflammation and secondary neuronal damage.

## INTRODUCTION

Neutrophil extracellular traps (NETs) are complexes released by neutrophils, consisting of DNA scaffolds associated with proteins such as citrullinated histone 3 (CitH3) and neutrophil elastase [1]. As a key component of innate immunity, NETs function to immobilize and eliminate diverse pathogens, including bacteria [1], fungi [2], viruses [3], and parasites [4]. However, accumulating evidence indicates that NET formation also occurs in noninfectious conditions, such as tumors [5-7], autoimmune disorders [8-10], and cardiovascular and cerebrovascular diseases [11-14]. NET formation is initiated by the activation of neutrophil protein-arginine deiminase type 4 (PAD4), which catalyzes histone citrullination. This process facilitates the release of neutrophil elastase and myeloperoxidase from azurophilic granules into the cytoplasm, where they translocate to the nucleus [15, 16]. Subsequent chromatin decondensation, driven by enzymatic modifications, results in the rupture of the nuclear envelope and extrusion of protein-decorated chromatin into the extracellular space, leading to NET release [17].

Neutrophils have been shown to migrate to sites of injury and accumulate within the microvasculature of ischemic and infarcted brain tissue [18]. Autopsy studies of patients with ischemic stroke have demonstrated that neutrophils can cross the vascular barrier and infiltrate the brain parenchyma [19]. Animal studies have further confirmed that neutrophils within the brain generate NETs following stroke [19-23]. These structures are predominantly localized to the perivascular space, perivascular brain parenchyma, and vascular lumen within infarcted regions [19]. NETs contribute to post-stroke pathology by activating the stimulator of interferon genes (STING)-TANK-binding kinase 1 signaling pathway, which compromises blood-brain barrier integrity and impairs angiogenesis [23]. Moreover, inhibition of NET formation significantly reduces microglial accumulation in infarcted areas [21], underscoring the role of NETs in amplifying inflammatory responses after stroke.

Absent in melanoma 2 (AIM2) is a DNA recognition protein belonging to the interferon-inducible HIN-200 protein family [24], [25]. Upon recognizing double-stranded DNA, AIM2 binds to the precursor of caspase-1 via apoptosis-associated speck-like protein containing a CARD (ASC) to form a complex and activate caspase-1. Activated caspase-1 mediates the cleavage of gasdermin D (GSDMD), generating an N-terminal fragment (N-GSDMD) that integrates into the cell membrane, forming pores that disrupt cellular integrity [26]. Additionally, activated caspase-1 cleaves the precursor of interleukin-1β (IL-1β) and interleukin-18 (IL-18), yielding mature IL-18 and IL-1β that are released by the cell [27]. These molecular events culminate in pyroptosis, a lytic and proinflammatory mode of cell death that exacerbates tissue damage in inflammatory conditions [28, 29].

Previous study has demonstrated that NETs activate the AIM2 inflammasome, leading to pyroptosis in alveolar macrophages [30]. In psoriasis, NET-driven AIM2 activation promotes the production of IL-1β, contributing to chronic inflammation [31]. In systemic lupus erythematosus, renal biopsy findings indicate that AIM2 interacts with NETs, preventing their degradation and potentially sustaining inflammatory responses [32]. More recently, NETosis in vascular plaques has been shown to trigger AIM2 inflammasome activation, promoting plaque inflammation, destabilization, and potential stroke recurrence [33]. Despite these findings, the role of NETs in post-stroke neuroinflammation and their interaction with the AIM2 inflammasome in brain tissue remain poorly understood. This study investigated whether NETs contribute to cerebral injury by activating the AIM2 inflammasome and inducing pyroptosis in brain tissue following stroke. Using an experimental stroke model and *in vitro* BV2 microglial cultures, we demonstrated that NETs promoted AIM2 inflammasome activation in microglia, induced pyroptotic cell death, and exacerbated stroke-related brain injury.

## MATERIALS AND METHODS

### Ethics statement and animals

This study was approved by the Ethics Committee of Sir Run Run Shaw Hospital, Zhejiang University School of Medicine (approval No. 20210309-34, 20220320). All participants provided written informed consent. All animal experiments were performed according to the Guide for the Care and Use of Laboratory Animals of the National Institutes of Health, and all procedures were approved by the Institutional Animal Ethics Committee of Sir Run Run Shaw Hospital of Zhejiang University. Eight-week-old male C57BL/6 mice were used in this study. All mice underwent an acclimatization period of at least 3 days before the experiments. Measures were taken to minimize the number of mice used and to reduce pain and distress.

### Clinical sample collection

Patients with acute ischemic stroke undergoing mechanical thrombectomy were prospectively enrolled between December 2020 and December 2021. Diagnosis and treatment were conducted by experienced neurologists. Patients were included in the analysis only if venous, femoral arterial, and infarct site arterial blood samples were successfully obtained. Exclusion criteria included the presence of an active infectious disease or a history of central nervous system disorders within the preceding six months. For sample collection, 2 mL of venous blood was drawn from each patient before treatment using EDTA-coated tubes. An additional 2 mL of femoral artery blood was obtained immediately after successful femoral artery puncture, while 2 mL of arterial blood from the infarct site was collected during the thrombectomy procedure. All blood samples were immediately stored at 4 ℃ and centrifuged at 3 000 rpm for 5 min at 4 ℃ to separate plasma within 2 h. The resulting plasma samples were preserved at −80 ℃ for NET quantification. Stroke severity was assessed using the National Institutes of Health Stroke Scale (NIHSS) at admission and 24 h post-procedure. Early neurological changes were evaluated based on the 24-h NIHSS score difference (NIHSS_baseline_ − NIHSS_24h_).

### NET quantification

NET levels in human plasma were quantified by detecting CitH3-DNA complexes, following established protocols [34, 35]. Briefly, 96-well black plates were coated with 5 μg/mL anti-CitH3 antibody (ab5103; Abcam) to capture CitH3 from plasma samples, which were incubated overnight at 4 °C. The wells were then blocked with freshly prepared 5% bovine serum albumin (BSA) for 2 h at room temperature (200 μL per well), followed by three washes with 200 μL PBS. Samples (100 μL) were added to each well, with two PBS wells serving as negative controls. After incubation for 2 h at room temperature, the wells were washed three times with 200 μL of PBS per well. DNA bound to CitH3 was quantified using a Quant-iT PicoGreen Kit (P7589; Invitrogen) following the manufacturer’s instructions. Briefly, 100 μL of diluted PicoGreen solution was added to each well and incubated on a shaker for 3 min at room temperature. Fluorescence was measured using a microplate reader (Molecular Devices, CA, USA) with an excitation wavelength of 480 nm and an emission wavelength of 520 nm. A standard curve was constructed based on DNA standards, and CitH3-DNA content in plasma samples was calculated according to the standard curve.

### Transient middle cerebral artery occlusion stroke (tMCAO) model

A tMCAO model was established in mice using the intraluminal filament technique [36]. Briefly, mice were anesthetized with 1.5% isoflurane and placed on an operating table. A midline cervical incision was made to expose the left carotid vasculature. The common carotid artery was ligated, and a silicone-coated monofilament was introduced through the external carotid artery into the internal carotid artery. The monofilament was advanced until it reached the origin of the middle cerebral artery, effectively occluding blood flow. After 1 h of ischemia, the monofilament was withdrawn, and the common carotid artery was reopened to restore cerebral perfusion. Sham-operated mice underwent identical procedures without monofilament insertion. Throughout the surgery, rectal temperature was maintained at 37.0 ± 0.5 ℃, and mice were placed on a heating pad postoperatively until full recovery.

To inhibit NET generation, GSK484, a selective PAD4 inhibitor, was administered based on previous research [13, 37, 38]. Briefly, a stock solution was prepared by dissolving GSK484 (17488, Cayman) in 99.9% ethanol at a concentration of 25 mg/mL, followed by dilution in sterile saline at a 1:50 ratio. Mice received intraperitoneal injections of GSK484 (4 mg/kg/day) for five consecutive days, with tMCAO performed on the third day of treatment. Moreover, NETs were degraded by intravenous injection of DNase I (10104159001, Roche) at a dose of 2.5 mg/kg, immediately before stroke induction, as previously described [39]. Vehicle controls were prepared identically, omitting GSK484 or DNase I.

For AIM2 knockdown in whole brain tissue, 3 μL of lentivirus-eGFP-shAIM2 (LV-AIM2, 3×10^8^ transduction units (TU)/mL; Obio Technology) was injected into the lateral ventricle 3 days prior to the establishment of tMCAO. To achieve microglia-specific AIM2 knock-down, 3 μL of adeno-associated virus (MG1.2)-Iba1-shAIM2-eGFP (AAV-AIM2, 3 ×10^8^ TU/mL; GeneChem) was administered into the lateral ventricle 1 month prior to the establishment of tMCAO. Empty vector control viruses (LV-NC and AAV-NC) were used as experimental controls. Viral injections were performed using a stereotaxic apparatus with the following coordinates relative to the bregma: anteroposterior (AP), −0.3 mm; mediolateral (ML), 1.0 mm; dorsoventral (DV), −2.6 mm.

### Western blot analysis

The peri-infarct region was defined as a 1-mm zone surrounding the infarct boundary, as described previously [40]. Cortical tissue from this region was collected, homogenized (Fig. 2A), and subjected to total protein extraction and quantification. Proteins were separated by sodium dodecyl sulfate-polyacrylamide gel electrophoresis (SDS-PAGE) and transferred to polyvinylidene fluoride (PVDF) membranes. To prevent nonspecific binding, membranes were blocked with 5% skim milk. Primary antibody incubation was performed overnight at 4 °C with rat anti-lymphocyte antigen-6 (Ly6G; 1:1 000, 127602; BioLegend), mouse anti-β-actin (1:1 000, 20536-1-AP; Proteintech), rabbit anti-citrullinated histone H3 (CitH3, 1:1 000, ab5103; Abcam), rabbit anti-histone H3 (H3, 1:1 000, 9715S; Cell Signaling Technology), mouse anti-AIM2 (1:1 000, sc-293174; Santa Cruz Biotechnology, Santa Cruz), rabbit anti-ASC (1:1 000, 67824S; Cell Signaling Technology), mouse anti-caspase-1 (1:1 000, AG-20B-0042-C100; AdipoGen), rabbit anti-GSDMD (1:1 000, ab219800; Abcam), rabbit anti-interleukin (IL)-1β (1:1 000, 12426S; Cell Signaling Technology), rabbit anti-IL-18 (1:1 000, 57058S; Cell Signaling Technology), rabbit anti-NLRP1 (1:500, A16212; ABclonal), rabbit anti-NLRP3 (1:1 000, 30109; Proteintech), rabbit anti-NLRC4 (1:200, A7382; ABclonal), and mouse anti-β-tubulin (1:5 000, FD0064; Fdbio Science). The membranes were then incubated with appropriate horseradish peroxidase-conjugated secondary antibodies (1:5 000, anti-rat (SA00001-10), anti-mouse (SA00001-1), anti-rabbit (SA00001-2); Proteintech) at room temperature for 1 h. Protein signals were detected using the ChemiDoc™ Touch Imaging System (Bio-Rad, CA, USA) and quantified using Image J software. Histone H3, β-actin, and β-tubulin were used as reference proteins for relative expression analyses.

### Isolation of neutrophils and NETs

Blood samples were collected from mice, and neutrophils were immediately isolated using a Histopaque density gradient separation method (10771, 11191; Sigma-Aldrich), as described previously [13]. Briefly, Histopaque 1077 (3 mL) was carefully layered onto Histopaque 1119 (3 mL) in a 15-mL conical centrifuge tube. The whole blood (1 mL) was then gently overlaid onto the uppermost layer of Histopaque 1077, and the gradient was centrifuged at 400 × *g* for 30 min at room temperature. Neutrophils were collected from the interface between the Histopaque 1077 and Histopaque 1119 layers. Subsequently, the isolated neutrophils were washed with 10 mL of phosphate-buffered saline (PBS) and centrifuged at 500 × *g* for 10 min at 4 ℃. The resulting pellet was resuspended in a hypotonic lysis buffer (R1010, Solarbio) to lyse residual erythrocytes. After additional centrifugation at 500 × *g* for 10 min at 4 ℃, neutrophils were resuspended in RPMI 1640 supplemented with HEPES (22400089; Gibco) for further experiments.

NET isolation was performed as previously described [41]. In brief, neutrophils (1 × 10^6^/mL) were seeded into culture plates and incubated at 37 ℃ in a 5% CO_2_ environment. After 1 h, neutrophils were stimulated with 100 nM phorbol myristate acetate (PMA) to induce NET formation. Following 4 h of incubation, the culture medium containing neutrophils and NETs was collected, resuspended in PBS, and centrifuged at 450 × *g* for 10 min at 4 ℃ to remove intact cells. The resulting supernatant was further centrifuged at 18 000 × *g* for 10 min at 4 ℃ to pellet NETs. Each NET-containing pellet was resuspended in Dulbecco’s Modified Eagle Medium (DMEM), and NET concentration was quantified using a Quant-iT PicoGreen dsDNA Assay Kit.

### Cell culture and treatment

BV2 microglial cells were cultured in DMEM supplemented with 10% fetal bovine serum. AIM2 knockdown was achieved by transducing BV2 cells with LV-AIM2 in the presence of 5 μg/mL polybrene, using an empty vector lentivirus (LV-NC) as a control. The multiplicity of infection (MOI) was set at 80. To assess the effect of NETs on pyroptosis, BV2 cells were exposed to purified NETs (300 ng/mL) for 12 h, with untreated cells serving as controls.

### Immunofluorescence staining

Mice were deeply anesthetized and transcardially perfused with PBS followed by 4% paraformaldehyde. Whole brains were extracted, post-fixed, and sectioned into 30 μm-thick coronal slices using a freezing microtome. Tissue sections were incubated with primary antibodies targeting key cellular markers, including goat anti-CD31 (1:200, AF3628; R&D Systems), rabbit anti-CitH3 (1:1 000, ab5103; Abcam), rat anti-Ly6G (1:200, 127602; BioLegend), rabbit anti-Iba1 (1:500, 10904-1-AP; Proteintech), mouse anti-Iba1 (1:200, RT1316; Huabio), rabbit anti-NeuN; 1:1 000, ab177487; Abcam), rabbit anti-GFAP, 1:500, 16825-1-AP; Proteintech), and mouse anti-AIM2 (1:1 000, sc-293174; Santa Cruz Biotechnology). Following primary antibody incubation, sections were treated with appropriate secondary antibodies, including Alexa Fluor 647-conjugated donkey anti-rabbit IgG (1:500, ab150075; Abcam), Alexa Fluor 488-conjugated donkey anti-mouse IgG (1:500, ab150153; Abcam), Cy3-conjugated donkey anti-goat IgG (1:100, SA00009-3; Proteintech), Alexa Fluor 488-conjugated goat anti-rabbit IgG (1:500, ab150077; Abcam), and Alexa Fluor 594-conjugated goat anti-mouse IgG (1:500, ab150080; Abcam). Cell nuclei were counterstained with 4’,6-diamidino-2-phenylindole (DAPI). Negative controls were prepared by omitting primary antibodies. Imaging was performed using confocal microscopy (TCS SP8; Leica).

BV2 cells were seeded onto glass-bottomed culture dishes and fixed with 4% paraformaldehyde following treatment. Immunostaining was performed using mouse anti-AIM2 (1:1 000, sc-293174, Santa Cruz Biotechnology) and rabbit anti-GSDMD (1:1 000, ab219800; Abcam) as primary antibodies. Secondary antibodies included CoraLite594-conjugated donkey anti-mouse IgG (1:200, SA00013-7; Proteintech) and Alexa Fluor 647-conjugated donkey anti-rabbit IgG (1:500, ab150075; Abcam). Cell nuclei were counterstained with DAPI. Negative controls were processed in parallel without primary antibodies. Confocal fluorescence imaging was conducted to visualize stained cells.

### Enzyme-linked immunosorbent assay (ELISA)

The levels of IL-1β and IL-18 in BV2 supernatants were measured using commercial ELISA kits (E-EL-M0037c and E-EL-M0730c; Elabscience) according to the manufacturer’s instructions. Briefly, 100 μL of each sample or standard was added to a 96-well plate and incubated at 37 °C for 90 min. After discarding the liquid, 100 μL of biotinylated detection antibody working solution was added, and the plate was incubated at 37 °C for 60 min. The wells were washed three times with PBS, and 100 μL of horseradish peroxidase-conjugated working solution was added to each well, followed by incubation at 37 °C for 30 min. After discarding the liquid, the plate was washed five times with PBS. Subsequently, 90 μL of substrate solution was added to each well, followed by incubation at 37 °C for 15 min. Finally, 50 μL of stop solution was added to each well, and absorbance was measured at 450 nm using a microplate reader. A standard curve was constructed based on the absorbance of the standards, and the concentration of the samples was calculated from the standard curve.

### Neurological function assessment

Neurological function was evaluated 3 days after tMCAO using a modified neurological severity score [42] (mNSS) on a 3–18 scale, where higher scores indicate better functional outcomes. The assessment comprised six domains: spontaneous activity, limb symmetry during movement, forepaw outstretching, climbing ability, body proprioception, and responses to vibrissae stimulation.

Motor coordination was evaluated using an accelerated rotarod test [43]. Mice underwent training for three consecutive days to maintain balance on a rotarod rotating at 4 rpm. Three days post-tMCAO, mice were placed on an accelerating rotarod that increased from 4 to 40 rpm over 5 min, and the latency to fall was recorded as an indicator of motor performance.

### Cerebral infarct volume quantification

Mice subjected to neurological function assessment were also used for infarct volume quantification. Three days after tMCAO, mice were sacrificed, and their brains were rapidly dissected and sectioned into 2-mm-thick coronal slices. The tissue sections were subsequently incubated in 2% 2,3,5-triphenyl-tetrazolium chloride (TTC, Sigma-Aldrich) at 37 ℃ for 10 min. Infarcted tissue appeared white, whereas healthy tissue stained red. Infarct volume was quantified by analyzing tissue images using ImageJ software, with calculations based on the infarct area in each section.

**Figure 1.**
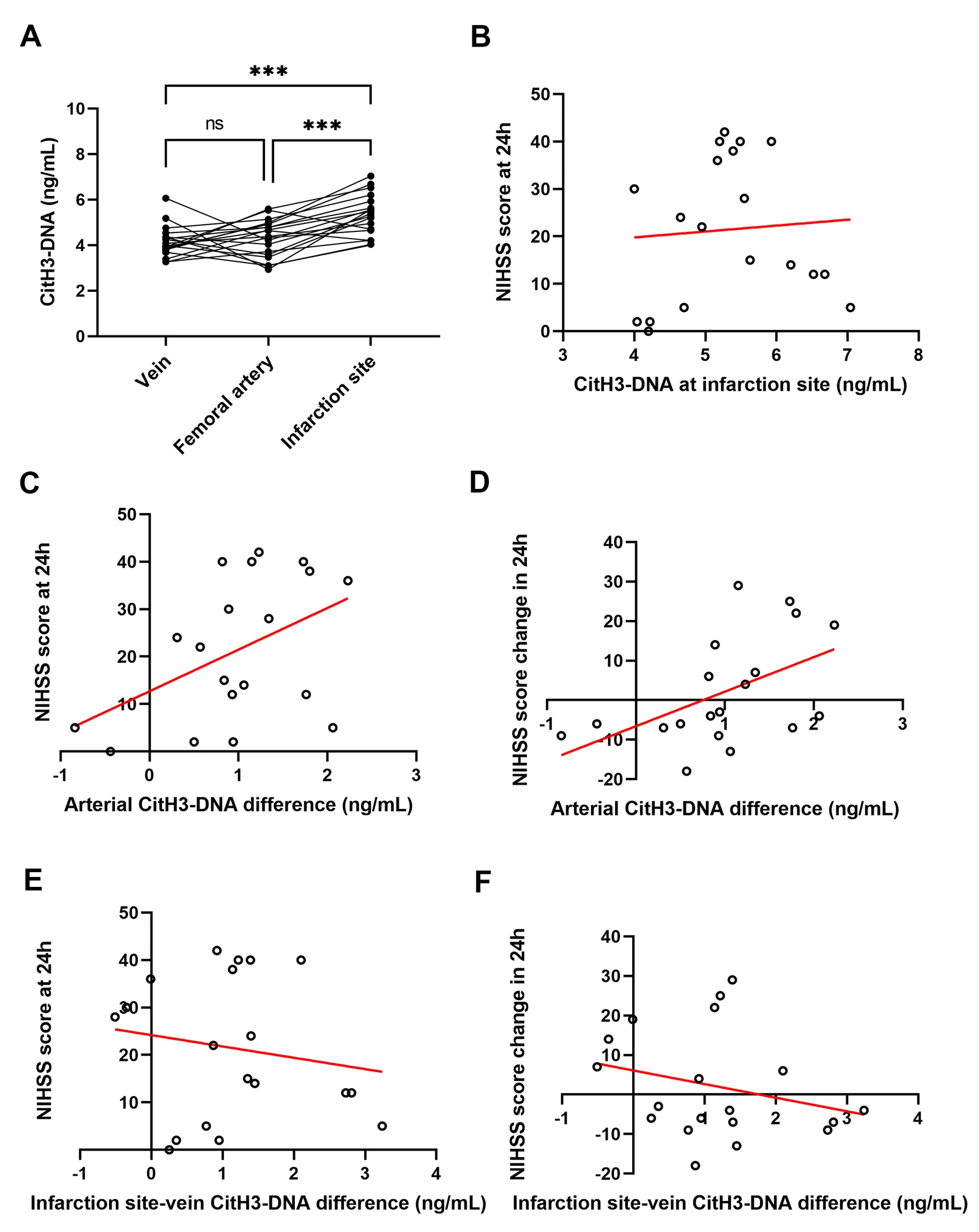
**NET levels in plasma from stroke patients undergoing thrombectomy.** (**A**) NET content was quantified in plasma collected from different vascular sites by measuring CitH3-DNA complexes (NETs) (*n* = 19). Statistical significance was determined using one-way ANOVA followed by Bonferroni’s correction. ***P < 0.001, ns, not significant. (**B**) Correlation between NIHSS score at 24 h and CitH3-DNA levels at infarct site (*r* = 0.07, *P* = 0.76, *n* = 19). (**C**) Correlation between NIHSS score at 24 h and arterial CitH3-DNA difference (*r* = 0.46, *P* = 0.05, *n* = 19). (**D**) Correlation between NIHSS score at 24 h and arterial CitH3-DNA difference (*r* = −0.16, *P* = 0.50, *n* = 19). (**E**) Correlation between NIHSS score change over 24 h and arterial CitH3-DNA difference (*r* = 0.50, *P* = 0.03, *n* = 19). (**F**) Correlation between NIHSS score change over 24 h and infarction site-to-vein CitH3-DNA difference (*r* = −0.26, *P* = 0.29, *n* = 19). Pearson’s correlation test was used for statistical analysis (B–F).

### Statistical analysis

Statistical analyses were performed using GraphPad Prism v9.0.0 software, with data presented as mean ± standard error of the mean (SEM). Normality was assessed using the Shapiro-Wilk test. For normally distributed data, comparisons between two groups were performed using an unpaired *t*-test, whereas comparisons among three or more groups were performed using one-way analysis of variance (ANOVA) followed by Bonferroni’s correction. For non-normally distributed data, non-parametric tests, including the Mann-Whitney and Kruskal-Wallis tests, were employed. If the sample size was insufficient for a reliable normality assessment (N < 6), non-parametric tests were used by default. Correlations between normally distributed continuous variables were assessed using Pearson’s correlation test, while Spearman’s correlation test was applied for non-normally distributed variables. Statistical significance was defined as *P* < 0.05.

**Figure 2.**
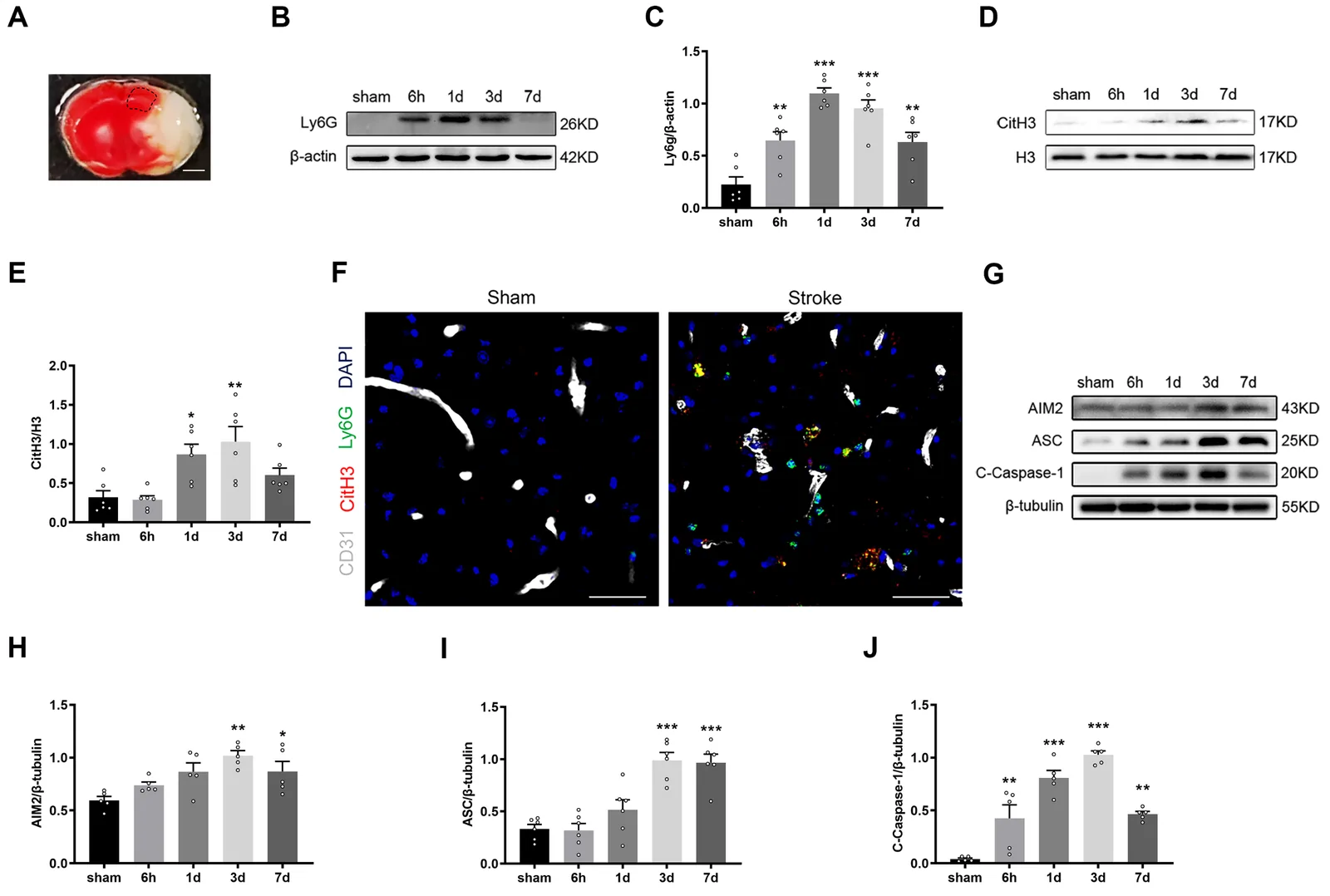
**NET formation and AIM2 inflammasome expression following stroke.** (**A**) Dashed lines indicate the peri-infarct region used for western blot and immunofluorescence staining. Scale bar, 1 mm. (**B–E**) Representative western blot bands and quantification of Ly6G and CitH3 protein levels in brain tissue at indicated timepoints after tMCAO (*n* = 6). Statistical significance was determined using one-way ANOVA followed by Bonferroni’s correction. (**F**) Representative immunofluorescence images showing NET structures (Ly6G+CitH3) and vessels (CD31) (*n* = 3). Scale bar, 100 μm. (**G–J**) Representative western blot bands and quantification of AIM2, ASC, and C-caspase-1 expression in brain tissue at indicated timepoints after tMCAO (*n* = 5–6). Statistical significance was determined using one-way ANOVA followed by Bonferroni’s correction (I) and Kruskal-Wallis test (H, J). Data are presented as mean ± SEM. *P < 0.05, **P < 0.01, ***P < 0.001 vs. sham.

## RESULTS

### NET formationn in peripheral blood at different sites in stroke patients

To quantify NET levels, CitH3-DNA complexes were measured in plasma samples from 19 stroke patients who underwent thrombectomy. The main clinical characteristics of these patients are summarized in Table 1. NET levels were significantly elevated in arterial blood from the infarct site compared to both venous and femoral arterial blood. However, no significant difference was observed between NET levels in venous and femoral arterial blood (Fig. 1A). No significant correlation was found between NET levels at the infarct site and NIHSS scores at 24 h (*n* = 19, *r* = 0.07, *P* = 0.76, Fig. 1B).

**Table 1:** Clinical characteristics of stroke patients (*n*=19).

Characteristics	Value
Male, *n* (%)	15 (78.9)
Age, mean ± SEM, y	70.1 ± 2.7
Hypertension, *n* (%)	14 (73.7)
Hypercholesterolemia, *n* (%)	7 (36.8)
Diabetes mellitus, *n* (%)	5 (26.3)
Smoking, *n* (%)	9 (47.4)
Previous stroke, *n* (%)	6 (31.6)
Coronary artery disease, *n* (%)	1 (5.3)
Atrial fibrillation, *n* (%)	9 (47.3)
Anterior circulation occlusion, *n* (%)	15 (78.9)
Cardioembolic stroke, *n* (%)	5 (26.3)
Baseline NIHSS, mean ± SEM	19.3 ± 2.4
IV t-PA, *n* (%)	8 (42.1)
TICl, 2c/3, *n* (%)	13 (68.4)

NIHSS, National Institute of Health Stroke Scale; IV, intravenous; t-PA, tissue-type plasminogen activator; TICI, thrombolysis in cerebral infarction.

**Figure 3.**
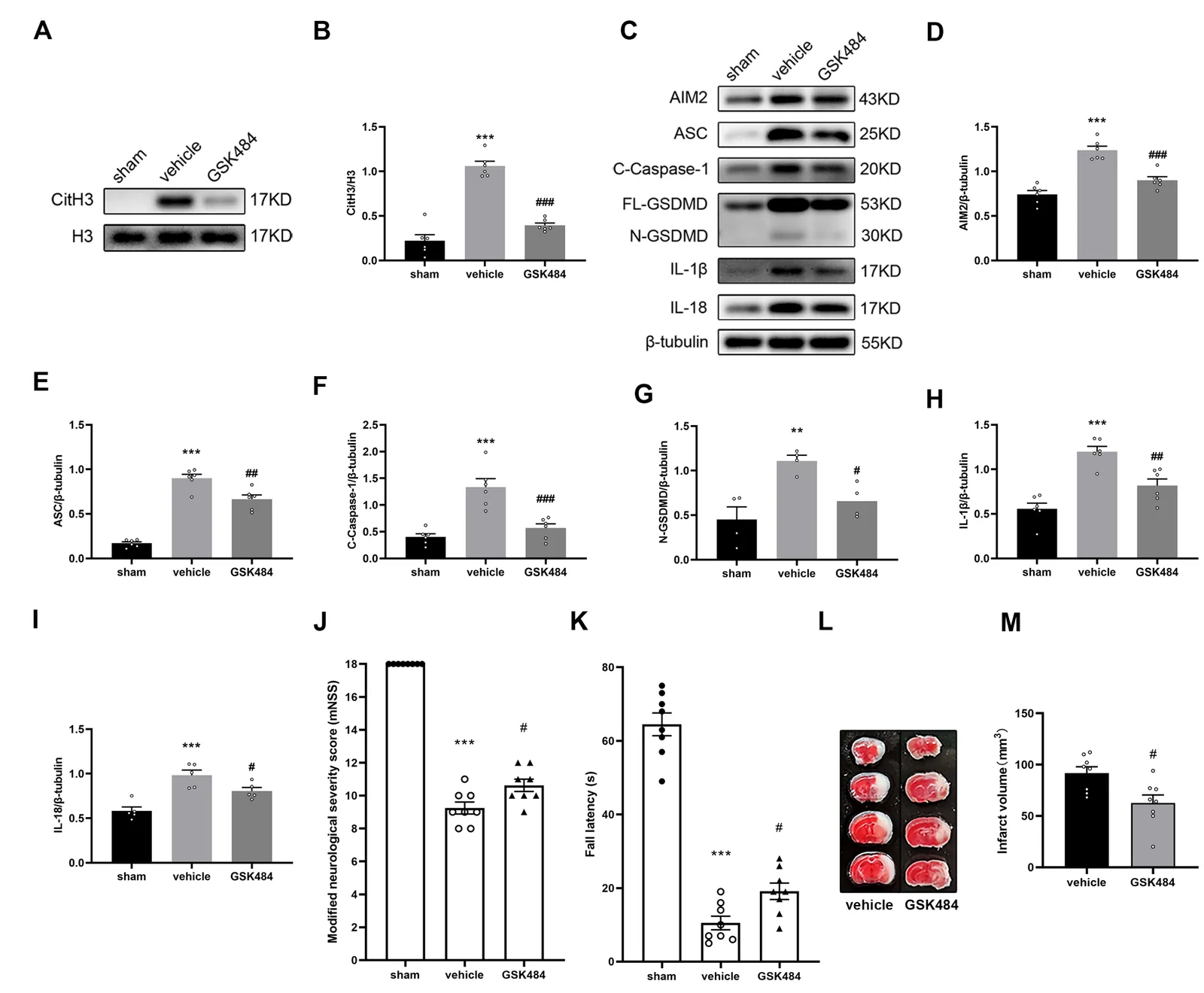
**Effects of GSK484 on AIM2 inflammasome activation, pyroptosis, and neurological injury following stroke**. (**A–B**) Representative western blot bands and quantification of CitH3 protein levels in brain tissue 3 days post-tMCAO following treatment with GSK484, a PAD4 inhibitor (*n* = 6). Statistical significance was determined using one-way ANOVA followed by Bonferroni’s correction.**(C–I)** Representative western blot bands and quantification of AIM2, ASC, C-caspase-1, N-GSDMD, IL-1β, and IL-18 protein levels in brain tissue 3 days post-tMCAO (*n* = 5–6). Statistical significance was determined using one-way ANOVA followed by Bonferroni’s correction (D-F, H) and Kruskal-Wallis test (G, I). (**J–K**) Neurological function assessment using mNSS and accelerated rotarod test 3 days post-tMCAO (*n* = 8). Statistical significance was determined using one-way ANOVA followed by Bonferroni’s correction. (**L–M**) Representative TTC-stained brain sections 3 days post-tMCAO and corresponding infarct volume quantification (*n* = 8). Statistical significance was determined using an unpaired two-tailed t-test. Data are presented as mean ± SEM. **P < 0.01, ***P < 0.001 vs. sham, #*P* < 0.05, ##*P* < 0.01, ###*P* < 0.001 vs. vehicle. FL-GSDMD: full-length GSDMD.

However, the difference in arterial CitH3-DNA levels between the infarct site and femoral artery (NETs_infarct site_ − NETs_femoral artery_) was positively correlated with both NIHSS scores at 24 h (*n* = 19, *r* = 0.46, *P* = 0.05; Fig. 1C) and NIHSS score changes over 24 h (NIHSS_baseline_ − NIHSS_24h_; *n*= 19, *r* = 0.50, *P* = 0.03; Fig. 1D). In contrast, neither NIHSS scores at 24 h (*n* = 19, *r* = −0.16, *P* = 0.50, Fig. 1E) nor NIHSS score changes over 24 h (*n* = 19, *r* = −0.26, *P* = 0.29, Fig. 1F) correlated with the difference in CitH3-DNA levels between the infarct site and venous blood (NETs_infarct site_ − NETs_vein_). These findings suggest that NET formation is associated with stroke severity, particularly in arterial blood from the infarct site.

### NET formation and AIM2 inflammasome activation in the brain following stroke

To investigate NET formation and AIM2 inflammasome activation in the peri-infarct region (Fig. 2A) after stroke, mice were subjected to tMCAO, and protein expression was assessed at multiple time points using western blot analysis. The levels of Ly6G, a neutrophil marker, and CitH3, a NET marker, were significantly elevated compared to the sham group. Ly6G expression peaked at 1 day post-tMCAO, while CitH3 expression reached its highest levels at 3 days (Fig. 2B–E). To determine the spatial relationship between NETs and cerebral vasculature, triple immunofluorescence staining was performed using Ly6G and CitH3 as NET markers and CD31 as a vascular marker 3 days post-tMCAO. NET structures were observed outside brain vessels, indicating extravascular NET formation (Fig. 2F). Western blot analysis was also used to assess AIM2 inflammasome activation by measuring the expression of AIM2, ASC, and cleaved caspase-1 (C-caspase-1). All three proteins exhibited significant up-regulation following tMCAO, peaking at 3 days post-stroke compared to sham controls (Fig. 2G–J).

**Figure 4.**
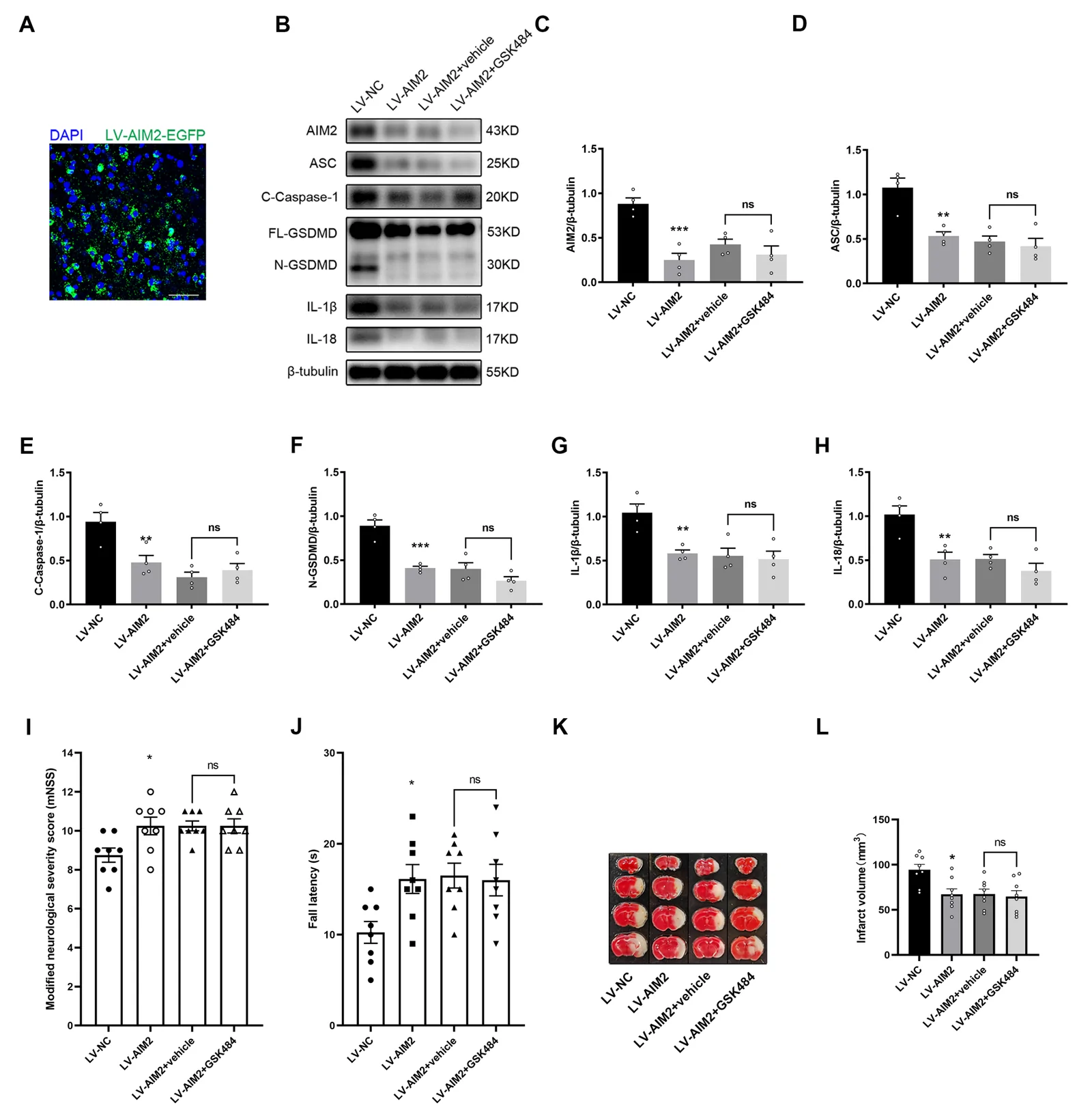
**Role of AIM2 in NET-induced pyroptosis.** (**A**) Schematic representation of LV-AIM2 transduction in the mouse brain. (**B–H**) Representative western blot bands and quantification of AIM2, ASC, C-caspase-1, N-GSDMD, IL-1β, and IL-18 protein levels 3 days post-tMCAO (*n* = 4). Statistical significance was determined using the Kruskal-Wallis test (G, I). (**I–J**) Neurological function assessment using mNSS and accelerated rotarod test 3 days post-tMCAO (*n* = 8). Statistical significance was determined using one-way ANOVA followed by Bonferroni’s correction.**(K–L**) Representative TTC-stained brain sections 3 days post-tMCAO and corresponding infarct volume quantification (*n* = 8). Statistical significance was determined using one-way ANOVA followed by Bonferroni’s correction. Data are presented as mean ± SEM. Scale bar, 50 μm, *P < 0.05, **P < 0.01, ***P < 0.001 vs. LV-NC.

### GSK484 inhibits AIM2-mediated pyroptosis after stroke

To evaluate the role of NET formation in inflammasome activation and pyroptosis, mice were treated with GSK484, a selective PAD4 inhibitor, for five consecutive days.

Western blot analysis demonstrated that GSK484 significantly reduced the expression of CitH3, AIM2, ASC, C-caspase-1, N-GSDMD, IL-1β, and IL-18 in brain tissue following tMCAO compared to the vehicle group (Fig. 3A–I). However, the expression of other inflammasomes were not affected by GSK484 in stroke mice (Supplementary Fig. 1). Consistent with the molecular finding of AIM2 inflammasome, GSK484-treated mice exhibited significant improvements in neurological function, as evidenced by lower mNSS scores and enhanced performance on the accelerated rotarod test compared to vehicle-treated mice (Fig. 3J–K). Furthermore, TTC staining revealed a significant reduction in infarct volume in GSK484-treated mice 3 days post-tMCAO relative to the vehicle group (Fig. 3L–M). Similar results were obtained when NETs were degraded with DNase I. DNase I reduced the expression of AIM2, ASC, C-caspase-1, N-GSDMD, IL-1β, and IL-18 (Supplementary Fig. 2A-G) and improved neurological function (Supplementary Fig. 2H-I).

**Figure 5.**
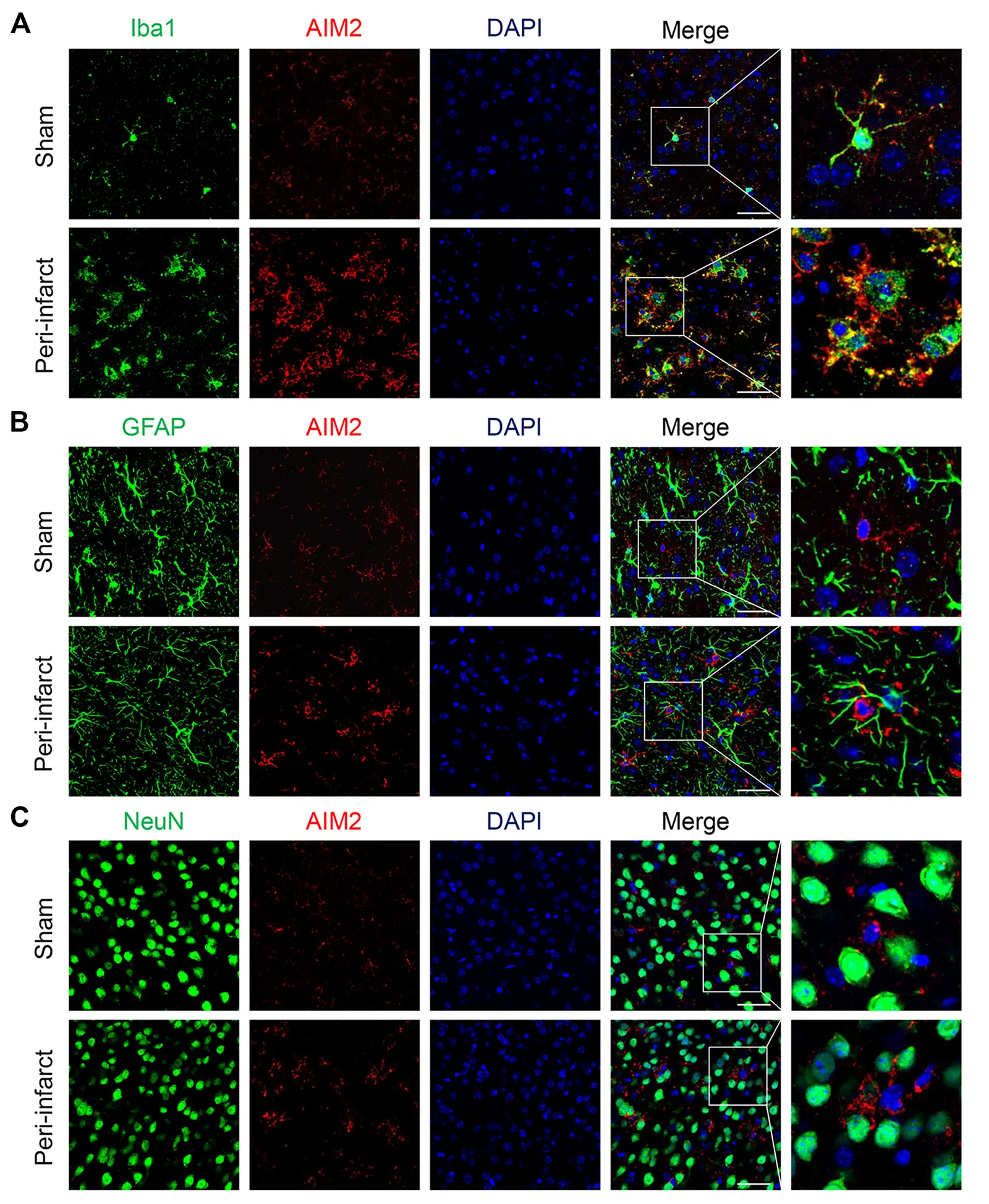
**Spatial expression of AIM2 in different brain cell types following stroke**. (**A–C**) Representative immunofluorescence images showing AIM2 colocalization with specific cell type markers (*n* = 3).**(A)** Double staining for AIM2 and Iba1 (microglia marker). (**B**) Double staining for AIM2 and GFAP (astrocyte marker). (**C**) Double staining for AIM2 and NeuN (neuronal marker). Scale bar = 50 μm. White boxed regions indicate magnified areas.

### NETs trigger AIM2-dependent pyroptosis following stroke

To investigate the role of NETs in AIM2-mediated pyroptosis, LV-AIM2 knockdown was performed via intracerebroventricular injection 3 days before tMCAO (Fig. 4A). Western blot analysis confirmed that LV-AIM2 significantly reduced the expression of AIM2, ASC, C-caspase-1, N-GSDMD, IL-1β, and IL-18 in brain tissue 3 days post-tMCAO compared to the LV-NC group (Fig. 4B–H). However, in mice receiving both LV-AIM2 and GSK484, these protein levels were not significantly lower than those treated with LV-AIM2 and vehicle (Fig. 4B–H). Neurological function was analyzed using the mNSS and accelerated rotarod test. Notably, mice in the LV-AIM2 group exhibited significant improvements in neurological outcomes compared to the LV-NC group. However, no additional enhancement in functional recovery was observed in LV-AIM2+GSK484-treated mice compared to those in the LV-AIM2+vehicle group (Fig. 4I–J). TTC staining was conducted to quantify infarct volume, which showed that infarct volume was significantly reduced in the LV-AIM2-treated mice compared to the LV-NC group. However, LV-AIM2+GSK484 treatment did not result in further infarct reduction relative to the LV-AIM2+vehicle group (Fig. 4K–L). These results demonstrate that NETs induce AIM2-associated pyroptosis.

### AIM2 is predominantly expressed in microglia following stroke

To determine the cellular distribution of AIM2 in the brain after stroke, double immunofluorescence staining was performed using AIM2 alongside markers for microglia, neurons, astrocytes, and endothelial cells. AIM2 expression was primarily localized to microglia within the peri-infarct region, with minimal to no detectable expression in neurons, astrocytes, or endothelial cells (Fig. 5A–C, Supplementary Fig. 3).

**Figure 6.**
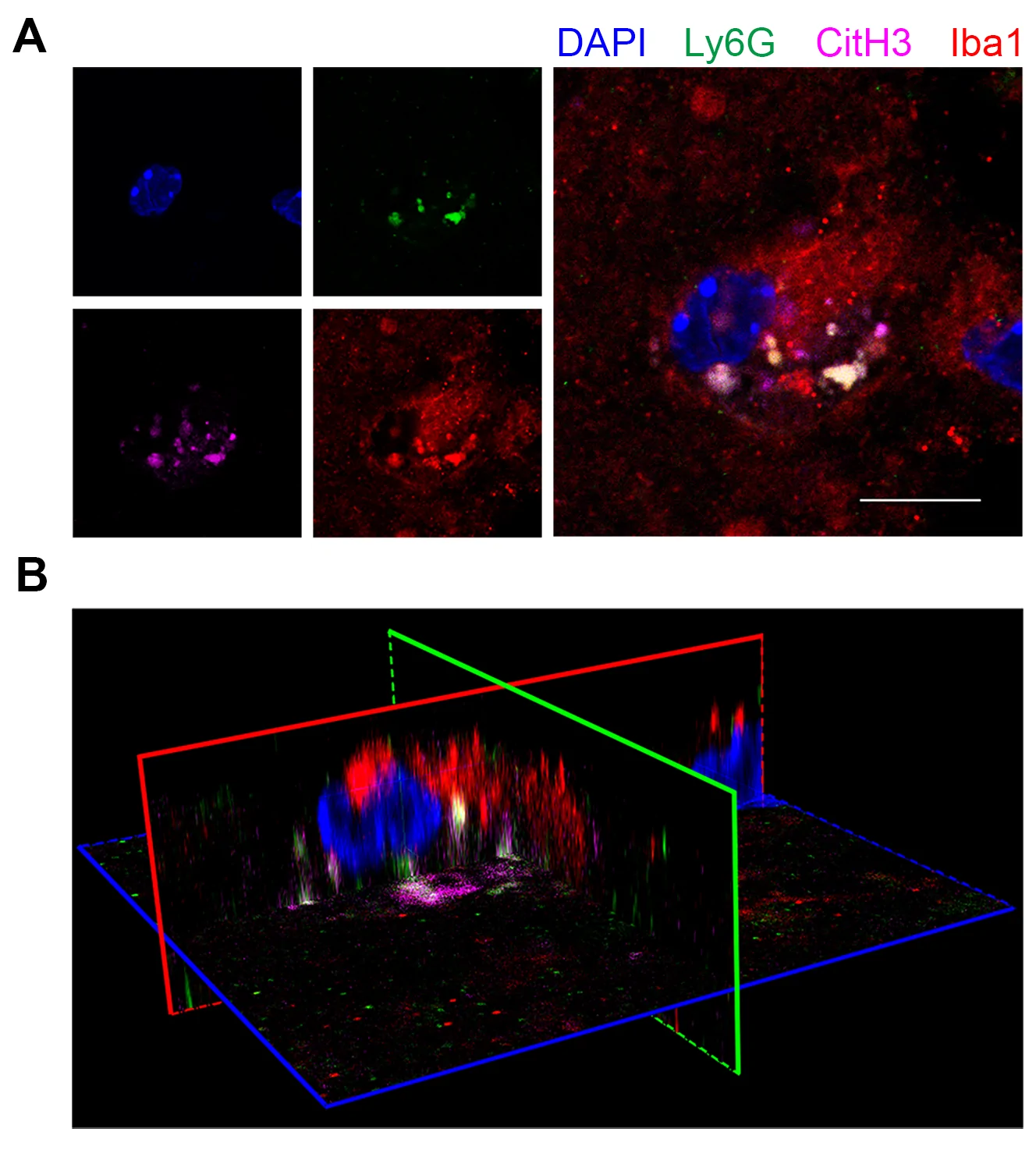
**Microglial phagocytosis of NETs following stroke.** (**A**) Representative immunofluorescence image showing triple staining for NET markers (CitH3 and Ly6G) and microglia marker (Iba1). (**B**) Representative cross-sectional view showing microglial engulfment of NETs. *n* = 3. Scale bar = 20 μm.

To further investigate the interaction between NETs and microglia, triple immunofluorescence staining was performed using markers for NETs (Ly6G+CitH3) and microglia (Iba1) 3 days post-tMCAO. The results revealed that microglia actively engulfed NETs (Fig. 6A–B).

### NETs induce AIM2-mediated pyroptosis in microglia *in vitro*

**Figure 7.**
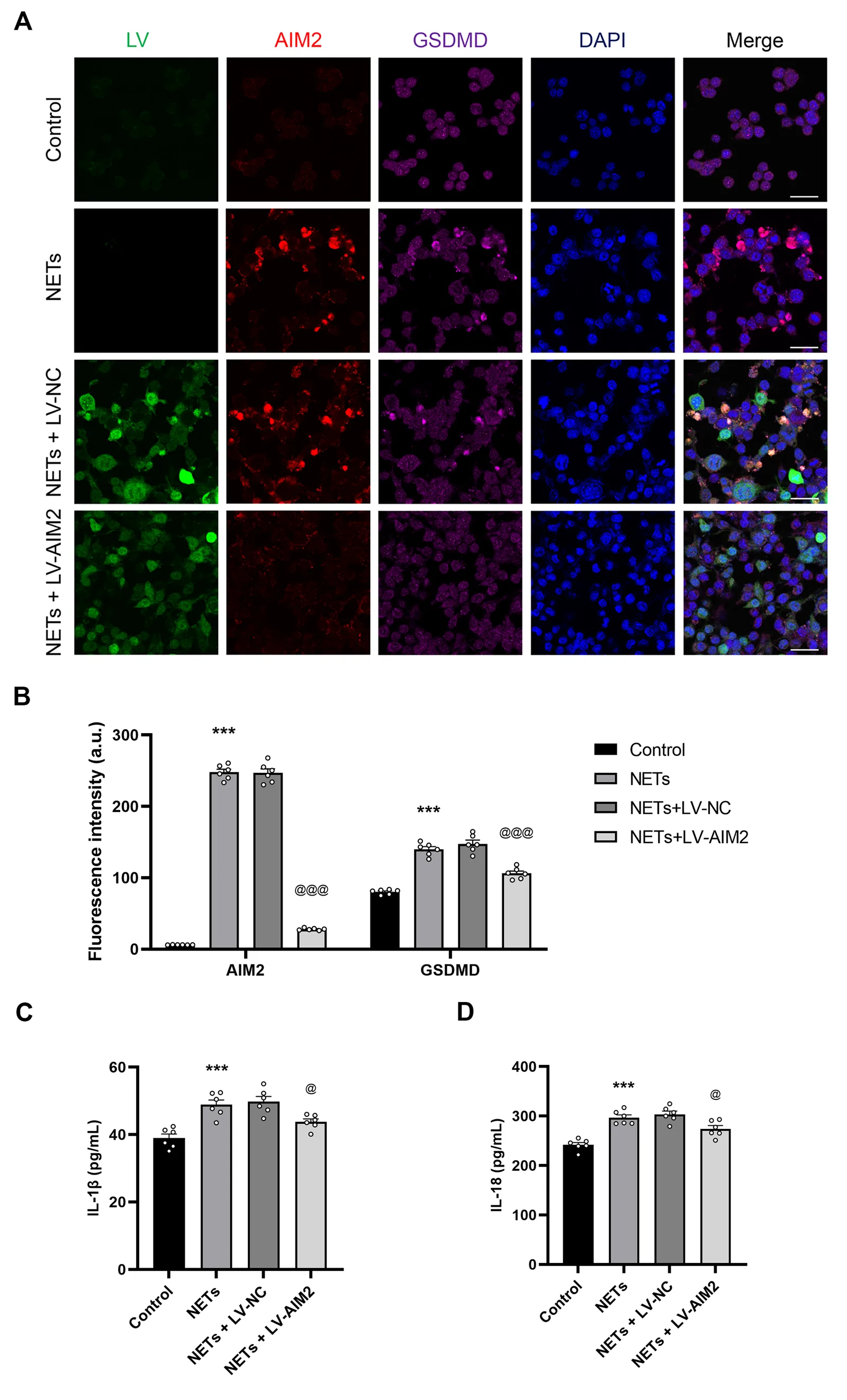
**Role of NETs in AIM2-associated pyroptosis in microglia *in vitro*.** (**A**) Representative immunofluorescence images of BV2 microglial cells following NET stimulation (*n* = 6). (**B**) Quantification of AIM2 and GSDMD fluorescence intensity (*n* = 6). Statistical significance was determined using one-way ANOVA followed by Bonferroni’s correction. (**C–D**) ELISA quantification of IL-1β and IL-18 levels in cell culture supernatants (*n* = 6). Statistical significance was determined using one-way ANOVA followed by Bonferroni’s correction. Scale bar = 50 μm. Data are presented as mean ± SEM. ***P < 0.001 vs. control, @*P* < 0.05, @@@*P* < 0.001 vs. NETs+LV-NC.

To confirm the effect of NETs on AIM2-dependent pyroptosis, BV2 microglial cells were cultured and exposed to purified NETs. Different concentrations of NETs were used to stimulate BV2 microglial cells for varying durations, and the IL-1β and IL-18 levels in the cell supernatant were measured. The results showed that stimulation with 300 ng/mL of NETs for 12 h significantly increased the concentrations of IL-1β and IL-18, whereas stimulation for 18 hours did not further elevate their levels (Supplementary Fig. 4A-B). Immunofluorescence staining also demonstrated that stimulation of BV2 microglial cells with 300 ng/mL of NETs for 12 h resulted in significant pyroptosis (Supplementary Fig. 4C). Based on these findings, subsequent experiments were conducted using 300 ng/mL NETs for 12 h of stimulation. Immunofluorescence staining demonstrated a marked up-regulation of AIM2 and GSDMD expression in NET-stimulated BV2 cells (Fig. 7A–B). Consistent with the *in vivo* findings, AIM2 knockdown via LV-AIM2 transduction significantly reduced NET-induced AIM2 and GSDMD expression in BV2 cells (Fig. 7A–B). Furthermore, ELISA analysis demonstrated that NET stimulation led to a significant increase in IL-1β and IL-18 levels in the supernatant, whereas AIM2 knockdown effectively attenuated this response (Fig. 7C–D).

### NETs drive AIM2-associated pyroptosis in microglia *in vivo* following stroke

**Figure 8.**
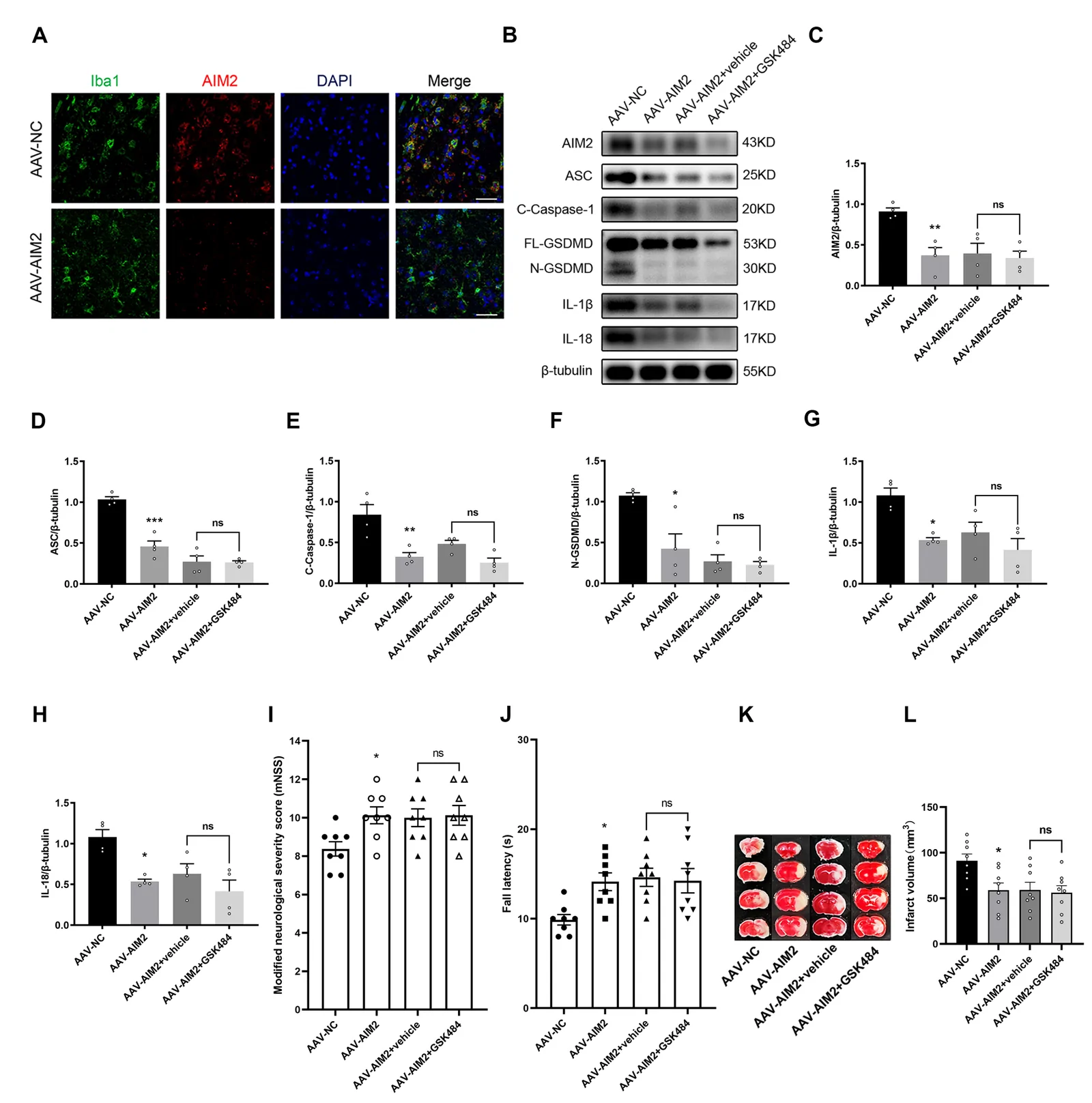
**Role of microglia AIM2 in NET-induced pyroptosis.** (**A**) Schematic representation of AAV-AIM2 transduction and its effect on AIM2 expression in the mouse brain post-tMCAO (*n* = 3). (**B–H**) Representative western blot bands and quantification of AIM2, ASC, C-caspase-1, N-GSDMD, IL-1β, and IL-18 protein levels 3 days post-tMCAO (*n* = 4). Statistical significance was determined using the Kruskal-Wallis test (G, I). (**I–J**) Neurological function assessment using the mNSS and accelerated rotarod test 3 days post-tMCAO (*n* = 8). Statistical significance was determined using one-way ANOVA followed by Bonferroni’s correction. (**K–L**) Representative TTC-stained brain sections 3 days post-tMCAO and corresponding infarct volume quantification (*n* = 8). Statistical significance was determined using one-way ANOVA followed by Bonferroni’s correction. Scale bar, 50 μm. Data are presented as mean ± SEM. *P < 0.05, **P < 0.01, ***P < 0.001 vs. AAV-NC.

To investigate the role of NETs in AIM2-dependent pyroptosis in microglia, AAV-AIM2, a microglia-specific knockdown vector, was administered 4 weeks before tMCAO to selectively suppress AIM2 expression (Fig. 8A). Western blot analysis confirmed that AAV-AIM2 effectively reduced the expression of AIM2, ASC, C-caspase-1, N-GSDMD, IL-1β, and IL-18 in brain tissue 3 days post-tMCAO compared to the AAV-NC controls (Fig. 8B–H). However, no additional reduction in these protein levels was observed in the AAV-AIM2+GSK484-treated mice compared to the AAV-AIM2+vehicle-treated mice (Fig. 8B–H). Consistently, behavioral assessments revealed that AAV-AIM2 treatment significantly improved neurological function, as indicated by mNSS and accelerated rotarod performance compared to the AAV-NC group. However, these functional improvements were not significantly greater in AAV-AIM2+GSK484-treated mice compared to the AAV-AIM2+vehicle group (Fig. 8I–J). TTC staining was conducted to assess infarct volume. AAV-AIM2 treatment significantly reduced infarct size compared to the AAV-NC group, but no further reduction was observed in AAV-AIM2+GSK484-treated mice relative to the AAV-AIM2+vehicle group (Fig. 8K–L). These findings indicate that NETs contribute to AIM2-dependent pyroptosis in microglia following stroke, and that AIM2 knockdown alone is sufficient to mitigate NET-induced neuroinflammation and ischemic injury.

## DISCUSSION

Neutrophil infiltration into brain tissue following stroke exacerbates inflammatory injury. While preclinical studies have demonstrated promising outcomes by inhibiting neutrophil infiltration [44]-[49], clinical trials have yielded disappointing results [50], [51], underscoring the need for alternative therapeutic strategies to mitigate post-stroke inflammation. This study explored the role of NETs, extracellular web-like structures formed by neutrophils, in pyroptotic cell death after stroke. Results showed that NET generation at the infarct site was associated with stroke severity and progression. Furthermore, NETs were generated within the brain parenchyma and contributed to neuronal injury through pyroptosis. Specifically, NETs induced pyroptosis in microglia via AIM2 inflammasome activation, with AIM2 knockdown effectively reducing pyroptotic cell death and alleviating brain injury.

Previous research on NETs in cardiovascular diseases has primarily focused on their role in atherosclerosis and myocardial infarction [11], [52]-[54]. Elevated NET markers have been detected in plasma collected from culprit lesions compared to femoral arterial blood in patients with ST-elevation acute coronary syndrome [54]. Expanding upon these findings, this study conducted a comprehensive analysis of NET levels in venous, femoral arterial, and infarct site arterial blood from stroke patients. Consistently, plasma NET levels were significantly higher in arterial blood from the infarct site than in femoral arterial or venous blood, while no significant difference was observed between femoral arterial and venous blood. Moreover, the change in NIHSS score over 24 h was correlated with the arterial CitH3-DNA difference, suggesting that increased NET formation at the infarct site contributes to stroke progression. To further explore the impact of NETs in stroke, a tMCAO model was established in mice. Western blot and immunofluorescence analyses confirmed neutrophil infiltration into brain tissue and NET formation within the parenchyma following stroke.

Previous research has demonstrated that neutrophils contribute to post-stroke brain injury, but their impact varies depending on the stroke model. Inhibiting or knocking out neutrophil elastase significantly reduces infarct volume in tMCAO mice but has no effect in permanent middle cerebral artery occlusion (pMCAO) models [55], suggesting that neutrophils play a more important role in ischemia-reperfusion injury than in permanent ischemia. This hypothesis is further supported by findings showing that NET inhibition does not significantly alter infarct volume in pMCAO mice [21]. Consistent with these observations, our study confirmed that NET formation exacerbated cerebral ischemia-reperfusion injury. Notably, NET accumulation in the ischemic brain worsened infarct volume in tMCAO mice, while pharmacological inhibition of NET formation with GSK484 reduced infarct volume and attenuated neurological deficits, reinforcing the pathological role of NETs in stroke, consistent with prior study [39].

AIM2 is a DNA recognition protein that assembles the inflammasome and induces pyroptosis upon activation. Previous studies have shown that the AIM2 inflammasome in macrophages can be triggered by herpes simplex virus 1 and poly(dA:dT), resulting in pyroptosis and the release of inflammatory mediators [29]. More recently, NETs—web-like chromatin structures rich in DNA—have been implicated in AIM2 inflammasome activation. NET-induced AIM2 activation has been observed in alveolar macrophages, where it promotes pyroptotic cell death [56]. Furthermore, AIM2 binds NETs *in vitro* and in systemic lupus erythematosus renal tissue, preventing NET degradation by DNase I [32]. However, the relationship between NETs and AIM2 activation in the brain remains unclear. This study demonstrated that AIM2 expression was elevated in peri-infarct brain tissue following stroke, and that NET inhibition suppressed AIM2 inflammasome activation. Moreover, targeting NETs not only reduced infarct volume but also improved neurological function in an AIM2-dependent manner. Immunofluorescence staining revealed that AIM2 was primarily localized in microglia. To further investigate the interaction between NETs and microglia, BV2 cells were exposed to NETs *in vitro*. Immunofluorescence analysis and ELISA confirmed that NET stimulation induced pyroptosis in BV2 cells. Selective AIM2 knockdown in microglia using an AAV approach effectively mitigated pyroptosis, improved neurological outcomes, and decreased infarct volume. These findings indicate that NETs exacerbate post-stroke brain injury by activating the AIM2 inflammasome in microglia, leading to pyroptotic cell death and inflammation.

Our findings have important clinical implications. Currently, endovascular intervention is a primary treatment for acute ischemic stroke caused by large artery occlusion, with 78%–96% of patients with anterior circulation large artery occlusion achieving successful reperfusion through this approach [57]-[61]. However, reperfusion alone does not guarantee a favorable prognosis. The DIRECT-MT trial reported reperfusion rates of 79.4% for direct thrombectomy and 84.5% for bridging thrombectomy within 4.5 hours of stroke onset, yet the rates of favorable functional outcomes remained low at 36.4% and 36.8%, respectively [58]. These findings underscore the disconnect between high reperfusion rates and actual clinical recovery. Our study demonstrated that NET formation following ischemia-reperfusion promoted microglial AIM2 inflammasome activation, leading to pyroptosis and exacerbating post-stroke brain injury. Based on this, targeting NET formation or AIM2 activation at the time of hospital admission in stroke patients undergoing endovascular intervention may represent a novel therapeutic approach to improve prognosis. However, neutrophils play a crucial role in immune responses, and systemic NET inhibition may compromise infection resistance. Similarly, AIM2 plays a complex immunological role, selectively modulating AIM2 expression in microglia within the human brain remains a significant challenge. Further research is necessary to develop precise and safe therapeutic strategies that mitigate NET- and AIM2-mediated neuroinflammation without disrupting essential immune functions.

This study has several limitations. Firstly, while the AIM2 inflammasome is primarily localized in the cytoplasm and is activated by self-DNA damage or microbial invasion, the precise mechanism by which NETs activate the microglial AIM2 inflammasome remains unclear. Although the findings suggest a link between NETs and AIM2 activation in microglia, existing studies have not provided a definitive mechanistic explanation [62]. Further investigations are needed to elucidate the molecular pathways underlying this interaction. Secondly, AIM2 exhibits diverse and context-dependent functions. Our study showed that AIM2 was predominantly expressed in microglia within adult male mouse brain tissue. However, prior research has reported AIM2 expression in neurons, astrocytes, and microglia in the developing mouse brain at postnatal day 5 [63]. During neurodevelopment, AIM2 activation by DNA damage can induce pyroptosis, and inhibiting AIM2-dependent pyroptosis may lead to increased DNA damage and behavioral abnormalities [63]. These findings highlight the crucial role of AIM2 in central nervous system development. In addition, AIM2 activation in microglia has been shown to suppress Parkinson’s disease progression by inhibiting AKT signaling and interferon regulatory factor 3 phosphorylation [64]. The multifunctional nature of AIM2 underscores the need for further studies to establish its role in different pathological conditions. Thirdly, the study was conducted exclusively in young male mice, whereas stroke predominantly affects elderly individuals. Future research should evaluate the role of NETs in aged and female mice to determine whether sex and aging influence NET-mediated inflammation and stroke pathology. Additionally, comorbidities are highly prevalent among stroke patients and may influence disease progression and treatment responses. Investigating NET-related effects in stroke models with relevant comorbidities will provide a more comprehensive understanding of their clinical relevance.

This study demonstrated that neutrophils infiltrating the brain parenchyma from peripheral blood generated NETs, which activated the AIM2 inflammasome in microglia, leading to pyroptotic cell death and exacerbation of post-stroke brain injury. Targeting AIM2 may offer a potential strategy for attenuating NET-induced neuroinflammation and ischemic damage.

## Supplementary Materials

The Supplementary data can be found online at: www.aginganddisease.org/EN/10.14336/AD.2024.1733.

## Data Availability

The data supporting the findings of this study are available from the corresponding author upon reasonable request.
