# Supplementary material for: Neutrophil Extracellular Traps Promote AIM2-Dependent Microglial Pyroptosis Following Stroke
Source: Aging Dis. 2025 May 30;17(3):1616–33. doi: 10.14336/AD.2024.1733 (PMC13061539; doi:10.14336/AD.2024.1733)
Supplement: Supplementary file 1 — The Supplementary data can be found online at: www.aginganddisease.org/EN/10.14336/AD.2024.1733. [file AD-17-3-1616-s.pdf]

SUPPLEMENTARY DATA

Neutrophil Extracellular Traps Promote AIM2-Dependent Microglial Pyroptosis Following Stroke

Hanze Chen, Linhui Ni, Jinhua Zhang, Xu Zheng, Yigang Chen, Xing Jin, Beibei Hu, Xinxin Xu[3], Qiwen Tang, Shuang Li, Yonggang Hao, Shilong Sun, Chengbin He, Shuxia Cao, Xingyue Hu

© 2025. Chen H et al. Published online at http://www.aginganddisease.org/EN/10.14336/AD.2024.1733

SUPPLEMENTARY DATA

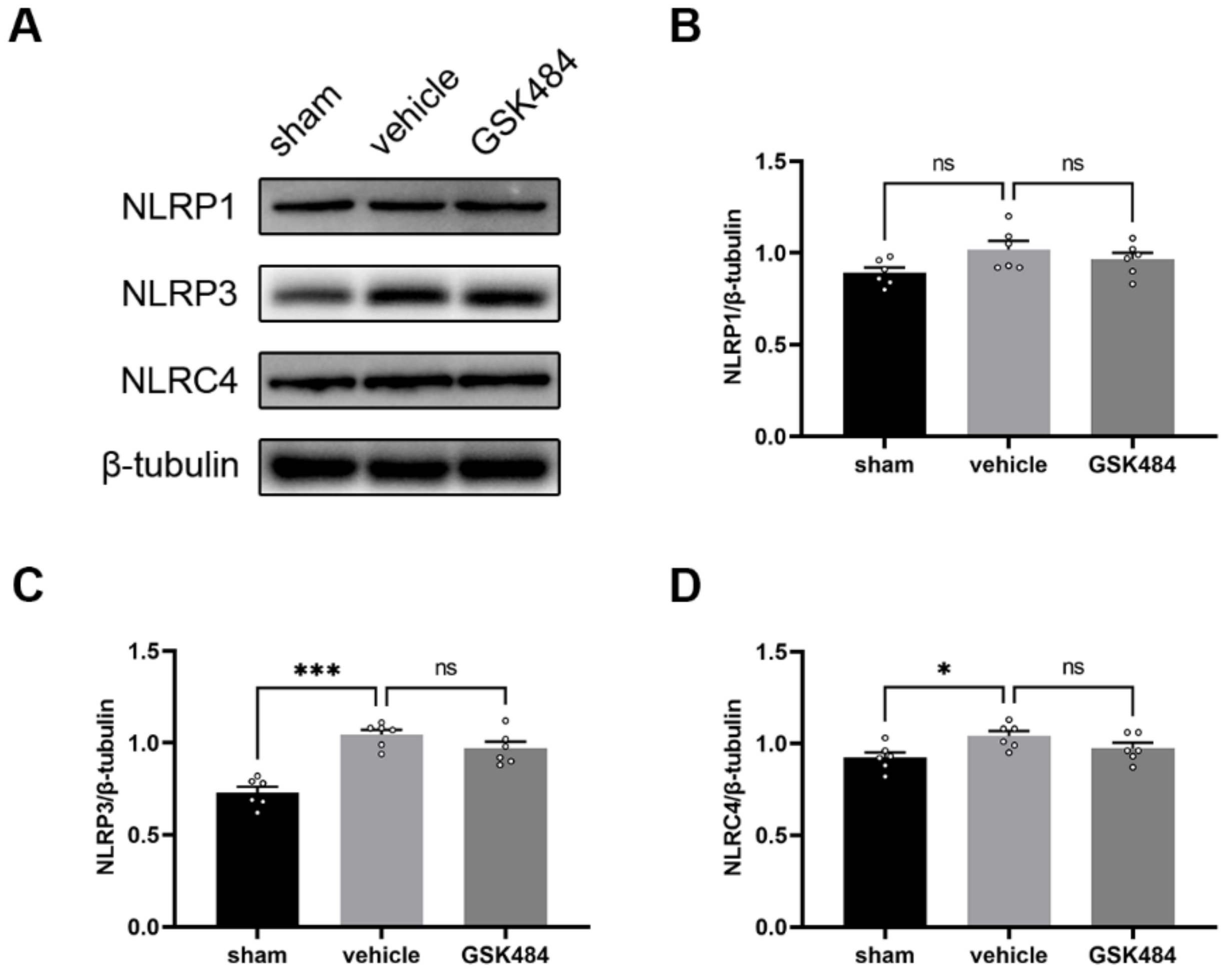

Supplementary Figure 1. Effects of GSK484 on inflammasomes following stroke. A–D Representative western blot bands and quantification of NLRP1, NLRP3, and NLRC4 protein levels in brain tissue 3 days post-tMCAO (n = 6). Statistical significance was determined using one-way ANOVA followed by Bonferroni's correction. Data are presented as mean ± SEM. ****P* < 0.001 vs. sham.

© 2025. Chen H et al. Published online at http://www.aginganddisease.org/EN/10.14336/AD.2024.1733

SUPPLEMENTARY DATA

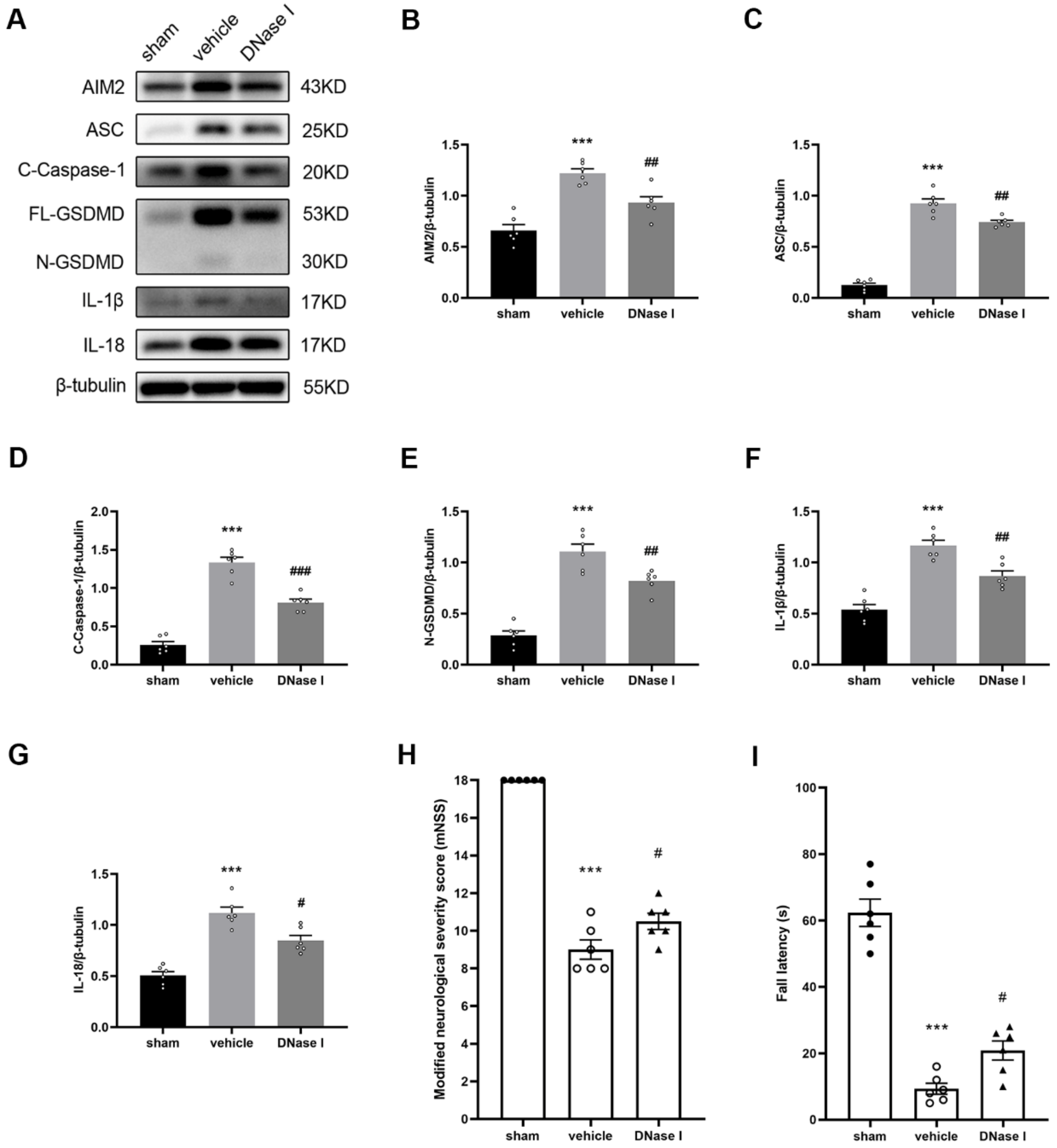

Supplementary Figure 2. Effects of DNase I on AIM2 inflammasome activation, pyroptosis, and neurological injury following stroke. A–G Representative western blot bands and quantification of AIM2, ASC, C-caspase-1, N-GSDMD, IL-1β, and IL-18 protein levels in brain tissue 3 days post-tMCAO (*n* = 6). Statistical significance was determined using one-way ANOVA followed by Bonferroni's correction. H–I Neurological function assessment using mNSS and accelerated rotarod test 3 days post-tMCAO (*n* = 6). Statistical significance was determined using one-way ANOVA followed by Bonferroni's correction. Data are presented as mean ± SEM. ***$P < 0.001$ vs. sham, #$P < 0.05$, ##$P < 0.01$, ###$P < 0.001$ vs. vehicle. FL-GSDMD: full-length GSDMD.

© 2025. Chen H et al. Published online at http://www.aginganddisease.org/EN/10.14336/AD.2024.1733

SUPPLEMENTARY DATA

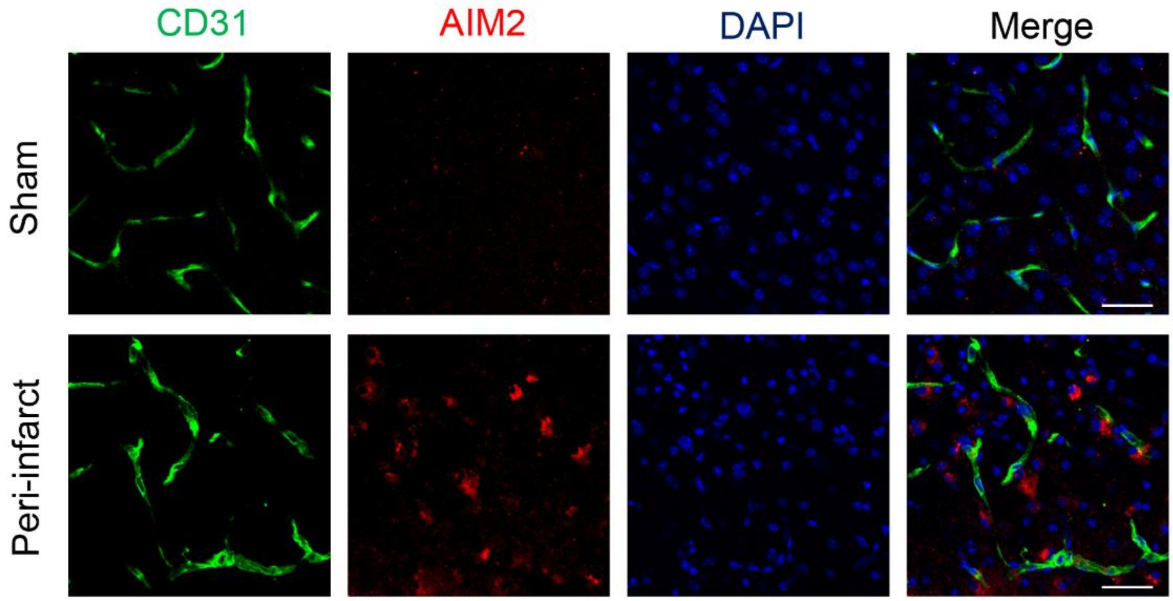

Supplementary Figure 3. Double staining of AIM2 and CD31. ***n*** **= 3. Scale bar = 50 µm.**

© 2025. Chen H et al. Published online at http://www.aginganddisease.org/EN/10.14336/AD.2024.1733

SUPPLEMENTARY DATA

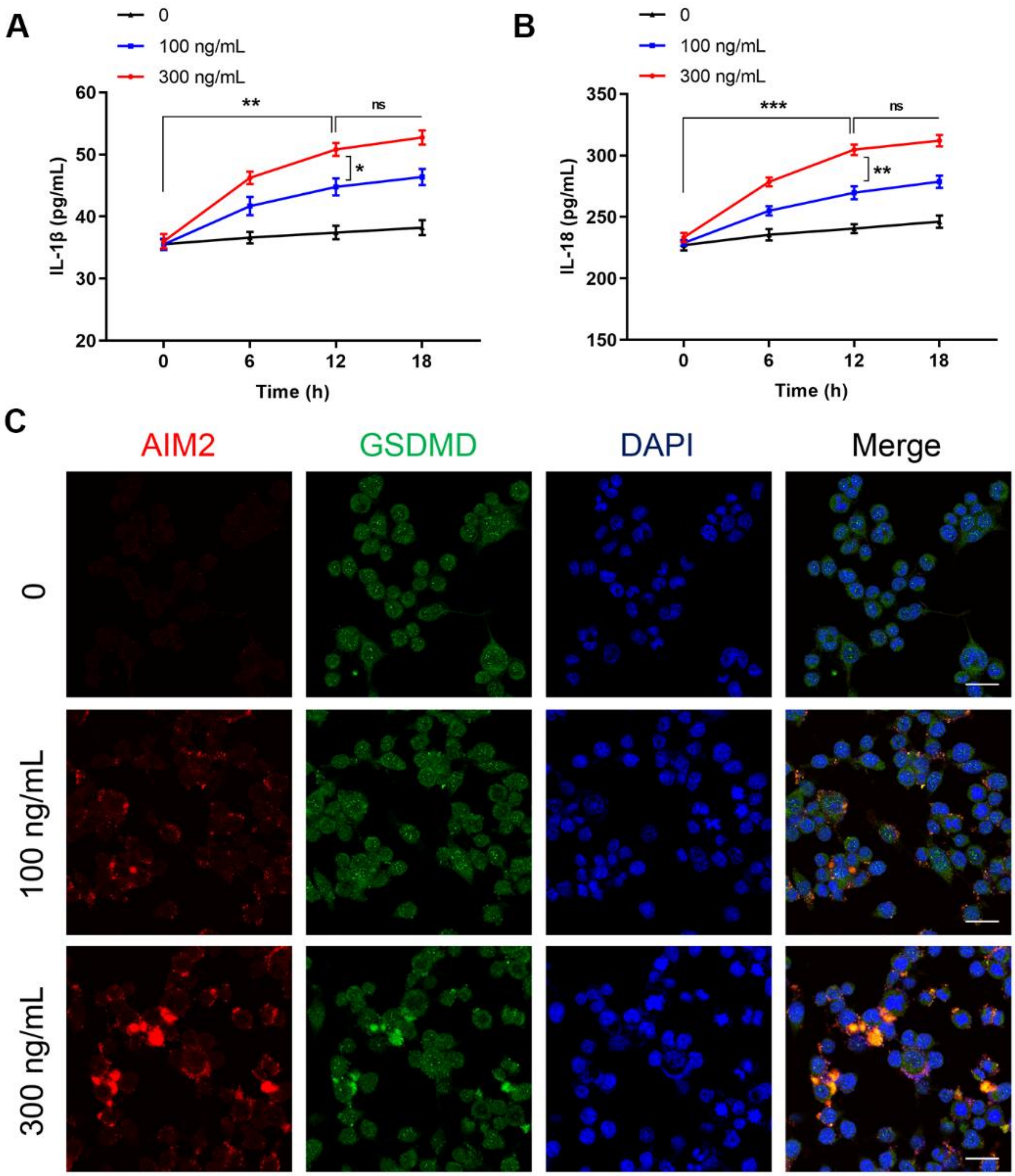

Supplementary Figure 4. NETs dose- and time-dependently induce AIM2-associated pyroptosis in microglia *in vitro*. A–B ELISA quantification of IL-1β and IL-18 levels in cell culture supernatants under different NET concentrations and stimulation durations. (n = 6). C Representative immunofluorescence images of BV2 microglial cells following stimulation with different concentrations of NET for 12 hours (n = 6). Statistical significance was determined using two-way ANOVA followed by Tukey's correction. Data are presented as mean ± SEM. *$P < 0.05$, **$P < 0.01$, ***$P < 0.001$.

© 2025. Chen H et al. Published online at http://www.aginganddisease.org/EN/10.14336/AD.2024.1733
